# Supplementary material for: Rats Orally Administered with Ethyl Alcohol for a Prolonged Time Show Histopathology of the Epididymis and Seminal Vesicle Together with Changes in the Luminal Metabolite Composition
Source: Biomedicines. 2024 May 3;12(5):1010. doi: 10.3390/biomedicines12051010 (PMC11117629; doi:10.3390/biomedicines12051010)
Supplement: Supplementary file 1 [file biomedicines-12-01010-s001.zip › biomedicines-2869294-supplementary.pdf]

Supplementary materials of the manuscript

"Rats orally administered with ethyl alcohol for a prolonged time show histopathology of the epididymis and seminal vesicle together with changes of the luminal metabolite composition"

by

Chayakorn Taoto, Nareelak Tangsrisakda, Wipawee Thukhammee,

Sitthichai Iamsaard, Jutarop Phetcharaburanin, Nongnuj Tanphaichitr

Supplementary Figures

Supplementary Figure S1. Hematoxylin/eosin-stained sections of the cauda epididymis from EtOH rats revealed marked disorientation and collapse of the epithelium with no discernment of the basement membrane in 10% of the total epithelium. Tissue fragments were also observed in the epididymal lumen of this highly deformed epithelium. The scale bar in the inset is 20 μm.

Supplementary Figure S2. Immunoblotting of the cauda epididymis and seminal vesicle with anti-caspase 9 antibody and anti-GAPDH antibody. The blot containing electrophoresed proteins from the cauda epididymis and seminal vesicle was cut between the MW of ~60,000 and 30,000, and this blot segment was used to probe with anti-caspase 9 and then reprobed with GADPH. This was to minimize the amount of antibodies needed for immunoblotting. The pro-caspase 9 band and cleaved-caspase 9 band as well as the GAPDH band in the first and second lanes were selected for presentation in Figure 5B. Similarly, for the presentation in Figure 6B, the pro-caspase 9 band and cleaved-caspase 9 band as well as the GAPDH band in lanes five and six were selected.

Supplementary Figure S3. Immunoblotting of the cauda epididymis and seminal vesicle with anti-caspase 3 antibody and anti-GAPDH antibody. The blot containing electrophoresed proteins from the cauda epididymis and seminal vesicle was cut between the MW of ~10,000 and ~35,000, and between the MW of ~35,000 and ~40,000. The first blot segment was used to probe with anti-caspase 3, whereas the second one was probed with GADPH. This was to minimize the amount of antibodies needed for immunoblotting. The pro-caspase 3 band and cleaved-caspase 3 band as well as the GAPDH band in the first and second lanes were selected for presentation in Figure 5C. Similarly, for the presentation in Figure 6C, the pro-caspase 3 band and cleaved-caspase 3 band as well as the GAPDH band in lanes five and six were selected.

Supplementary Figure S4. The S-plots derived from the altered metabolic profiling of the caudal epididymal fluid (CEF) in control versus EtOH-treated groups. The selected candidate variables with *p* value cut-off of |0.05| and *p*(corr) cut-off of |0.6| are colored in red. The metabolites with decreased levels after EtOH treatment included (1) carnitine, (2) myo-inositol, (3) fructose, (4) glycerophosphocholine (GPC), (5) alanine and (6) fructose 2,6-bisphosphate.

Supplementary Figure S5. The S-plots derived from the altered metabolic profiling of the seminal vesicle fluid (SVF) in control versus EtOH-treated groups. The selected candidate variables with *p* value cut-off of |0.05| and *p*(corr) cut-off of |0.6| are colored in red. The metabolites with decreased levels after EtOH treatment included (1) lactate, (2) glycerate, (3) citrate, (4) fructose, (5) myo-inositol, (6) leucine and (7) isoleucine.

Supplementary Tables

Supplementary Table S1. Identification of metabolites in the caudal epididymal fluid (CEF) based on their chemical shift values (ppm) from the website, https://hmdb.ca.

Supplementary Table S2. Identification of metabolites in the seminal vesicle fluid (SVF) based on their chemical shift values (ppm) from the website, https://hmdb.ca.

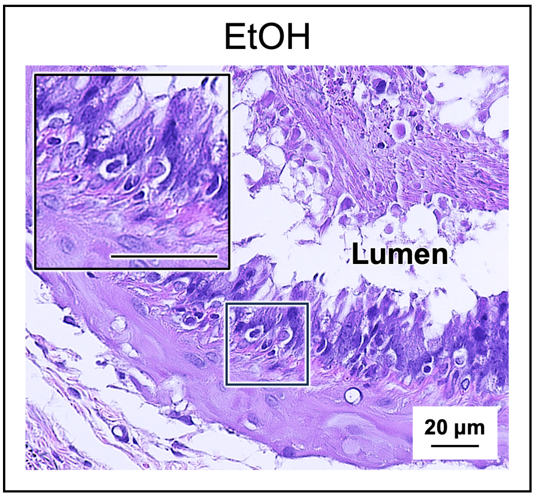

Supplementary Figure S1. Hematoxylin/eosin-stained sections of the cauda epididymis from EtOH rats revealed marked disorientation and collapse of the epithelium with no discernment of the basement membrane in 10% of the total epithelium. Tissue fragments were also observed in the epididymal lumen of this highly deformed epithelium. The scale bar in the inset is 20 µm.

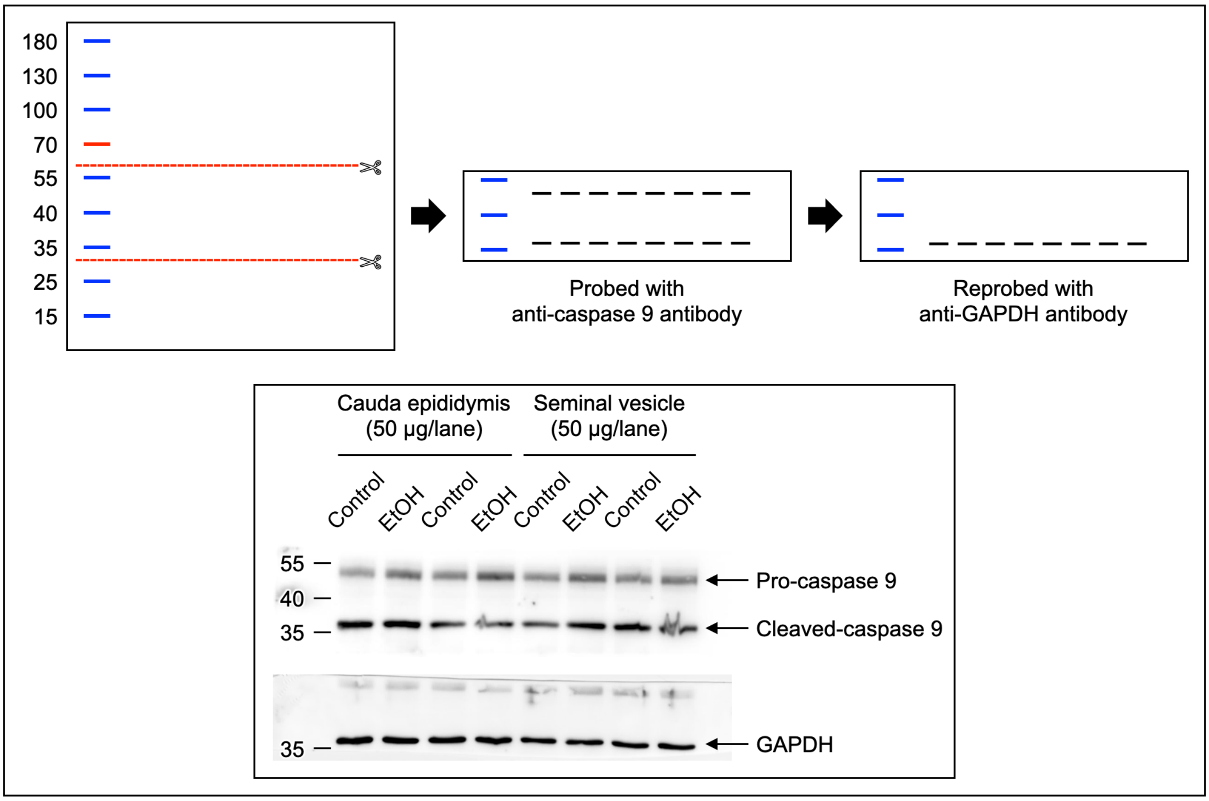

Supplementary Figure S2. Immunoblotting of the cauda epididymis and seminal vesicle with anti-caspase 9 antibody and anti-GAPDH antibody. The blot containing electrophoresed proteins from the cauda epididymis and seminal vesicle was cut between the MW of ~60,000 and 30,000, and this blot segment was used to probe with anti-caspase 9 and then reprobed with GADPH. This was to minimize the amount of antibodies needed for immunoblotting. The pro-caspase 9 band and cleaved-caspase 9 band as well as the GAPDH band in the first and second lanes were selected for presentation in Figure 5B. Similarly, for the presentation in Figure 6B, the pro-caspase 9 band and cleaved-caspase 9 band as well as the GAPDH band in lanes five and six were selected.

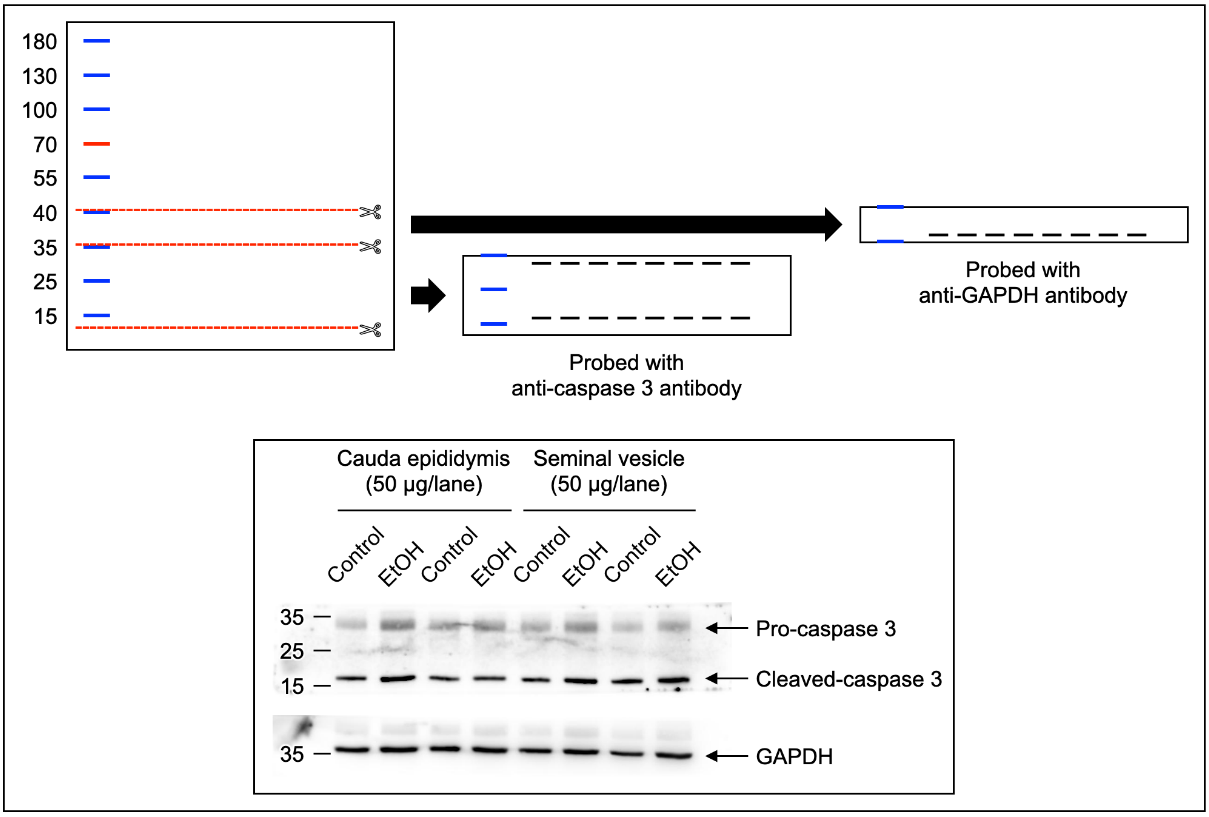

Supplementary Figure S3. Immunoblotting of the cauda epididymis and seminal vesicle with anti-caspase 3 antibody and anti-GAPDH antibody. The blot containing electrophoresed proteins from the cauda epididymis and seminal vesicle was cut between the MW of ~10,000 and ~35,000, and between the MW of ~35,000 and ~40,000. The first blot segment was used to probe with anti-caspase 3, whereas the second one was probed with GADPH. This was to minimize the amount of antibodies needed for immunoblotting. The pro-caspase 3 band and cleaved-caspase 3 band as well as the GAPDH band in the first and second lanes were selected for presentation in Figure 5C. Similarly, for the presentation in Figure 6C, the pro-caspase 3 band and cleaved-caspase 3 band as well as the GAPDH band in lanes five and six were selected.

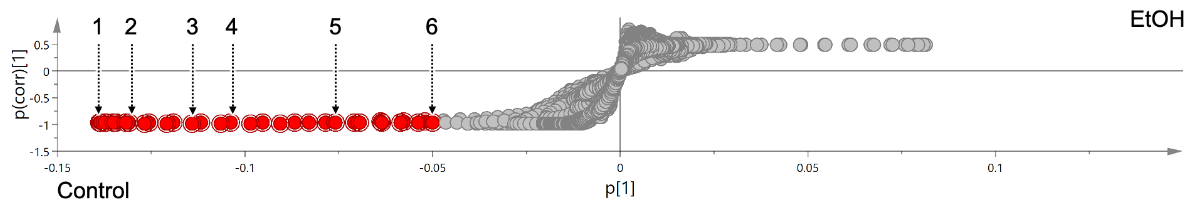

Supplementary Figure S4. The S-plots derived from the altered metabolic profiling of the caudal epididymal fluid (CEF) in control versus EtOH-treated groups. The selected candidate variables with *p* value cut-off of |0.05| and *p*(corr) cut-off of |0.6| are colored in red. The metabolites with decreased levels after EtOH treatment included (1) carnitine, (2) myo-inositol, (3) fructose, (4) glycerophosphocholine (GPC), (5) alanine and (6) fructose 2,6-bisphosphate.

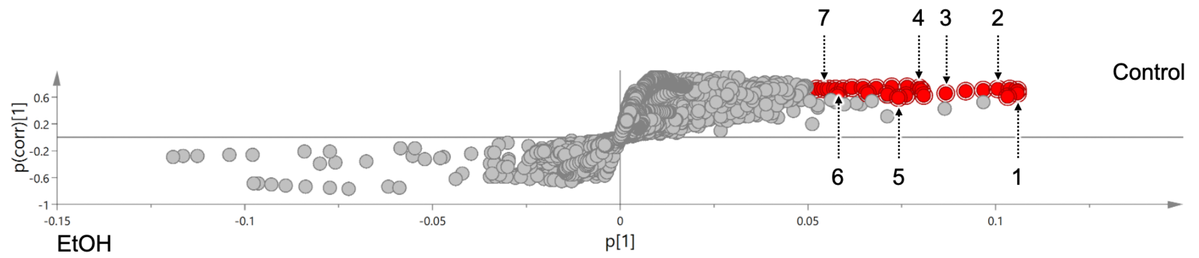

Supplementary Figure S5. The S-plots derived from the altered metabolic profiling of the seminal vesicle fluid (SVF) in control versus EtOH-treated groups. The selected candidate variables with *p* value cut-off of |0.05| and *p*(corr) cut-off of |0.6| are colored in red. The metabolites with decreased levels after EtOH treatment included (1) lactate, (2) glycerate, (3) citrate, (4) fructose, (5) myo-inositol, (6) leucine and (7) isoleucine.

Supplementary Table S1. Identification of metabolites in the caudal epididymal fluid (CEF) based on their chemical shift values (ppm) from the website, https://hmdb.ca.

No.	Chemical shift (ppm)	Multiplicity	STOCSY	Metabolite
1	1.32374	d*	1.32374 (d), 4.09023 (q)	Lactate
2	1.47547	d	1.47547 (d), 3.73798 (q)	Alanine
3	1.92124	s*	1.92124 (s)	Acetate
4	2.07701	s	2.07701 (s), 2.52312 (dd), 2.62405 (dd), 3.20709 (s), 3.60610 (d), 3.86852 (dd)	Acetylcarnitine
5	2.14699	s	2.14699 (s), 2.63414 (t), 3.86852 (t)	Methionine
6	2.42993	dd*	2.42993 (dd), 3.23333 (s), 3.42779 (m)	Carnitine
7	3.04156	s	3.04156 (s), 3.93412 (s)	Creatine
8	3.20709	s	3.20709 (s), 3.47960 (m), 4.06129 (m)	Choline
9	3.23333	s	3.23333 (s), 3.59634 (m), 3.86852 (m), 4.30621 (m)	Glycerophosphocholine

10	3.23333	s	3.23333 (s), 3.90586 (s)	Betaine
11	3.26126	s	3.26126 (s)	Trimethylamine N-oxide
12	3.28413	t*	3.28413 (t), 3.52199 (dd), 3.60610 (t), 4.06129 (t)	Myo-inositol
13	3.56337	d	3.56337 (d), 3.58692 (d), 3.65387 (m), 3.99367 (m)	Fructose
14	3.65387	q*	3.65387 (q), 3.90586 (m), 4.05120 (s)	Fructose 2,6-bisphosphate
15	8.19706	s	8.19706 (s), 8.21623 (s)	Adenine
16	8.46082	s	8.46082 (s)	Formate

s: singlet; d: doublet; t: triplet; q: quartet; dd: doublet of doublet.

Supplementary Table S2. Identification of metabolites in the seminal vesicle fluid (SVF) based on their chemical shift values (ppm) from the website, https://hmdb.ca.

No.	Chemical shift (ppm)	Multiplicity	STOCSY	Metabolite
1	0.89176	m*	0.89176 (m), 1.62586 (m), 3.70905 (t)	Leucine
2	0.92540	t*	0.92540 (t), 0.97318 (d), 1.21675 (m), 1.36613 (m), 2.18198 (m), 3.67271 (d)	Isoleucine
3	1.32374	d*	1.32374 (d), 4.08316 (q)	Lactate
4	2.09753	s*	2.09753 (s), 2.94366 (dd), 4.37283 (m)	Acetylcysteine
5	2.52279	d	2.52279 (d), 2.68130 (d)	Citrate
6	3.04123	s	3.04123 (s), 3.93412 (s)	Creatine
7	3.23333	s	3.23333 (s), 3.59601 (m), 3.85573 (m), 4.30554 (m)	Glycerophosphocholine
8	3.23333	s	3.23333 (s), 3.90586 (s)	Betaine

9	3.26092	t	3.26092 (t), 3.52266 (dd), 3.60610 (t), 4.06096 (t)	Myo-inositol
10	3.59601	d	3.59601 (d), 3.64008 (d), 3.80829 (d), 4.02294 (m), 4.06386 (m)	Fructose
11	3.70905	dd*	3.70905 (dd), 3.80829 (dd)	Glycerate
12	7.83538	s	7.83538 (s)	Xanthine

s: singlet; d: doublet; t: triplet; m: multiplet; dd: doublet of doublet.
